# Supplementary material for: Study protocol: a mixed-methods realist evaluation of the Universal Health Visiting Pathway in Scotland
Source: BMJ Open. 2020 Dec 30;10(12):e042305. doi: 10.1136/bmjopen-2020-042305 (PMC7780504; doi:10.1136/bmjopen-2020-042305)
Supplement: Supplementary data [file bmjopen-2020-042305supp001.pdf]

Supplementary file

Supplementary file 1. Health visitor and parent questionnaire topics

Health Visitor Questionnaire Topics

- The UHVP implementation process
- Overall views on the UHVP – including any added value and unintended consequences
- Training (views on the quality, relevance, frequency, gaps and CPD opportunities)
- Confidence to deliver all aspects of the UHVP
- Contacts with families (e.g., whether they are delivering all visits, assessments etc.)
- Relationships with families (e.g., has the UHVP affected health visitors' relationships with families?)
- Perceptions of how well the UHVP is working in relation to the identification of concerns relating to families/children
- Relationships with and referrals to other services (including working collaboratively, sharing information, and awareness of wider services etc.)
- Working with families who have complex additional needs
- IT systems (e.g. do they have access to the required systems, are the systems fit for purpose, do they manage, within the constraints of their workload, to complete the required monitoring of the UHVP?)
- Resources for parents (e.g. do health visitors feel they have all the necessary leaflets/materials to meet the needs of parents? What are their views on the quality of information in the materials etc.?)
- Impact of the UHVP on topics such as: keeping children safer; child development; child health and wellbeing; and positive health behaviours among parents.

Parent Questionnaire Topics

- Continuity of care from their Health Visitor
- Perceptions of their relationship with their Health Visitor
- What topics they have covered with their Health Visitor?
- How much they feel they know about specific health/childcare issues?
- Additional services their Health Visitor has suggested
- Confidence in own parenting
- Suggestions for improving the health visiting service
- Demographics – household structure, working status, gender, age, income, SIMD (Scottish Index of Multiple Deprivation) profile, indication of urbanity or rurality
