# Supplementary material for: Study protocol: a mixed-methods realist evaluation of the Universal Health Visiting Pathway in Scotland
Source: BMJ Open. 2020 Dec 30;10(12):e042305. doi: 10.1136/bmjopen-2020-042305 (PMC7780504; doi:10.1136/bmjopen-2020-042305)
Supplement: Author's manuscript [file bmjopen-2020-042305.draft_revisions.pdf]

BMJ Open is committed to open peer review. As part of this commitment we make the peer review history of every article we publish publicly available.

When an article is published we post the peer reviewers' comments and the authors' responses online. We also post the versions of the paper that were used during peer review. These are the versions that the peer review comments apply to.

The versions of the paper that follow are the versions that were submitted during the peer review process. They are not the versions of record or the final published versions. They should not be cited or distributed as the published version of this manuscript.

BMJ Open is an open access journal and the full, final, typeset and author-corrected version of record of the manuscript is available on our site with no access controls, subscription charges or pay-per-view fees (http://bmjopen.bmj.com).

If you have any questions on BMJ Open's open peer review process please email info.bmjopen@bmj.com

BMJ Open

Study protocol: A mixed-methods realist evaluation of the Universal Health Visiting Pathway in Scotland

Journal:	*BMJ Open*
Manuscript ID	bmjopen-2020-042305
Article Type:	Protocol
Date Submitted by the Author:	03-Jul-2020
Complete List of Authors:	Doi, Lawrence; University of Edinburgh, School of Health in Social Science<br>Morrison, Kathleen; University of Edinburgh, School of Health in Social Science<br>Astbury, Ruth ; University of West of Scotland, School of Health and Life Science<br>Eunson, Jane; Ipsos MORI Scotland<br>Horne, Margaret; University of Edinburgh Centre for Population Health Sciences,<br>Jepson, Ruth ; Scottish Collaboration for Public Health Research and Policy (SCPHRP)<br>Marryat, Louise; University of Edinburgh, Salvensen Mindroom Research Centre<br>Ormston, Rachel ; Ipsos MORI Scotland<br>Wood, Rachael; NHS National Services Scotland, Information Services Division; University of Edinburgh, Child Life and Health
Keywords:	PUBLIC HEALTH, Community child health < PAEDIATRICS, Health policy < HEALTH SERVICES ADMINISTRATION & MANAGEMENT, Maternal medicine < OBSTETRICS

SCHOLARONE™
Manuscripts

BMJ

I, the Submitting Author has the right to grant and does grant on behalf of all authors of the Work (as defined in the below author licence), an exclusive licence and/or a non-exclusive licence for contributions from authors who are: i) UK Crown employees; ii) where BMJ has agreed a CC-BY licence shall apply, and/or iii) in accordance with the terms applicable for US Federal Government officers or employees acting as part of their official duties; on a worldwide, perpetual, irrevocable, royalty-free basis to BMJ Publishing Group Ltd ("BMJ") its licensees and where the relevant Journal is co-owned by BMJ to the co-owners of the Journal, to publish the Work in this journal and any other BMJ products and to exploit all rights, as set out in our licence.

The Submitting Author accepts and understands that any supply made under these terms is made by BMJ to the Submitting Author unless you are acting as an employee on behalf of your employer or a postgraduate student of an affiliated institution which is paying any applicable article publishing charge ("APC") for Open Access articles. Where the Submitting Author wishes to make the Work available on an Open Access basis (and intends to pay the relevant APC), the terms of reuse of such Open Access shall be governed by a Creative Commons licence – details of these licences and which Creative Commons licence will apply to this Work are set out in our licence referred to above.

Other than as permitted in any relevant BMJ Author's Self Archiving Policies, I confirm this Work has not been accepted for publication elsewhere, is not being considered for publication elsewhere and does not duplicate material already published. I confirm all authors consent to publication of this Work and authorise the granting of this licence.

For peer review only

Study protocol: A mixed-methods realist evaluation of the Universal Health Visiting Pathway in Scotland

Lawrence Doi[1]*, Kathleen Morrison[1], Ruth Astbury[2], Jane Eunson[3], Margaret Horne[4], Ruth Jepson[1], Louise Marryat[4], Rachel Ormston[3], Rachael Wood[4]

*Correspondence: larry.doi@ed.ac.uk

[1]Scottish Collaboration for Public Health Research and Policy, School of Health in Social Science, Rm 1B31, 5 Forrest Hill, University of Edinburgh, Edinburgh, EH1 2QL

[2]School of Health and Life Science, University of West of Scotland, Paisley, PA1 2BE

[3]Ipsos MORI Scotland, 4 Wemyss Place, Edinburgh, EH3 6DH

[4]Salvensen Mindroom Research Centre, Kennedy Tower, Royal Edinburgh Hospital, University of Edinburgh, Edinburgh, EH10 5HF

For peer review only

ABSTRACT

Introduction

The growing political emphasis on the early years reflects the importance of these formative years of life. Health visitors in the UK are uniquely positioned to improve health outcomes for children and families and to reduce health inequalities. Recently, there has been a policy change in Scotland in an attempt to enhance the delivery of the universal health visiting service. This study aims to examine the extent to which the enhanced universal health visiting pathway is implemented and delivered across Scotland and to assess any associated impacts.

Methods and analysis

This is a mixed-methods study incorporating four methodological components and uses realist evaluation as the overall conceptual framework. The study comprises three phases (1) initial programme theory development; (2) programme theory validation; and (3) programme theory refinement. The programme theory validation will use interviews and focus groups data of parents and health visitors, and conduct case note review at five study sites. It also involves a national survey of parents and health visitors and routine data analysis of existing secondary data. The analyses of the ensuing qualitative and quantitative data will be carried out using a convergent mixed-methods approach to ensure continuous triangulation of multiple data. The findings of the evaluation will provide contextually relevant understanding of how the UHVP works and evidence the impact of increased investments in health visiting in Scotland.

Ethics and dissemination

This protocol has been approved by the School of Health in Social Science Research Ethics Committee, University of Edinburgh. Additional approvals have been granted by the Public Benefit and Privacy Panel for health and social care in Scotland for the case note review and survey elements of the evaluation. The findings will be prepared as reports to the funders and presented at conferences. It will be submitted for publication in peer-reviewed journals.

For peer review only

Strengths and limitations of this study
• This is a large study using a theory driven approach of realist evaluation to examine the implementation and delivery of a national universal health visiting pathway.<br>• The study is expected to generate contextually relevant understanding of how the universal health visiting pathway works and evidence the impact of increased investments in the programme, whilst identifying areas for improvement, which may be relevant to other similar programmes.<br>• Using mixed-methods would ensure that both qualitative and quantitative data are employed to provide continuous triangulation of data, thereby enhancing the credibility of the study findings.<br>• By not using well established experimental research approaches, some may argue against the robustness of the study design.

BACKGROUND

The formative early years, which lay the foundations for physical, intellectual and emotional development, have profound life-long implications for individual health and wellbeing, education, achievement and economic status[1]. Recent early years policies across the UK reflect this and have emphasised the need for greater prevention, early identification and intervention during the early-years stage (pre-birth to five years old). The early years, including child development, protection and welfare have been recognised as key public health priorities, which are crucial to reducing health inequalities across the life course[2]. Additionally, children who experience disadvantage in the early years are at a higher risk of injury, social, emotional and cognitive difficulties[3].

Health visitors have played a longstanding and vital role supporting children and families throughout the UK. Health visiting practice has been firmly rooted in public health for over 150 years, with overall goals of health promotion and disease prevention. The professional practice of health visiting comprised 'planned activities which aim to improve the physical, mental, emotional and social health and wellbeing of the population, preventing disease and reducing inequalities in health'[4]. Health visiting is a proactive service that searches for, and responds to, health needs at the individual, family, group and community level.

Since its inception, the health visiting profession has been heavily influenced by the policies of changing governments. Over the years, the pushing and pulling of various agendas have drastically altered the scope of the profession and the nature of the service it provides[5]. This is in part due to the fact the health visitors work with children, families and communities, areas where strong and often polarising political views are held. More so, health visitors are uniquely positioned to work across health and social care divides, which few other professions are able to do. This enables them to deliver a range of health promotion, intervention and illness prevention activities to individuals and families in a variety of settings.

The growing recognition for the importance of the early years, the current political emphasis on this and a growing evidence base for health visiting practice has led to large reinvestments in health visiting services across the UK. Government policies have sought to harness health visiting as a key asset to improve health outcomes for children under the age of five and to reduce health inequalities. Efforts have also been made nationally to recruit, train and employ more health visitors leading to increased recognition and re-definition of the health visiting profession as a whole.

In 2013, the Chief Nursing Officer's Directorate, Scottish Government, undertook a scoping exercise of health visiting practice in Scotland. The findings demonstrated that there was a significant degree of variation across the service in terms of assessment, resources and visiting patterns being delivered by health visitors to families in Scotland. A refocused approach to health visiting was published by the Scottish Government in 2013[6]. The changes took into account the changing policy landscape relating to the early years, children and families, and sought to ensure that workforce capability and capacity would be equipped to

For peer review only - http://bmjopen.bmj.com/site/about/guidelines.xhtml

successfully deliver these policies. Following substantial investment in the service, the Universal Health Visiting Pathway (UHVP) was introduced in 2015.

The UHVP refocuses the role of the health visitor and includes changes to caseload weighting and management; intervention delivery; education, training and resources; and visiting patterns. The aim of this study is to examine the extent to which the UHVP is implemented and delivered across Scotland and to assess any associated impacts. To achieve this, a robust mixed-methods realist evaluation proposal has been developed to understand 'what works for whom, why and in what circumstances'. Incorporating four methodological components, this paper presents a protocol describing the use and purpose of these methods over two stages of a national-level evaluation project.

For peer review only

METHODS

Conceptual framework

This is a mixed-methods study that uses realist evaluation as the overall conceptual framework to examine the UHVP across Scotland. Realist evaluation is increasingly utilised to evaluate nursing and healthcare programmes because of the valuable insight it provides into how such programmes work and how they can be improved[7]. Realist evaluations are well suited to addressing the complexity of healthcare systems and are able to produce useful findings for decision makers keen to improve healthcare programmes[8]. The strength of realist evaluation is that it can draw valuable lessons about how particular conditions make particular outcomes more likely[9]. Its goal is to explain and generate knowledge of how to improve a programme. It can also provide transferable lessons that may be used by others who intend to implement a similar programme elsewhere[10]. Realist evaluation argues that in order for the findings of evaluation to be useful to decision makers, there is the need to ask the question "what works for whom, why and in what circumstances?" By asking this question, the evaluation focuses on explaining how outcomes are achieved in respect to their contexts. This is particularly important because the UHVP has been implemented in the midst of significant policy developments in maternal and child health within Scotland, including the Family Nurse Partnership programme[11] and the Children and Young People (Scotland) Act 2014, which includes Getting it Right for Every Child (GIRFEC) and Named Person policies[12]. It is important that outcomes of the evaluation are sensitive to such policy contexts.

<Figure 1>

The study will evaluate the contexts, mechanisms, and outcomes of the UHVP and make recommendations for the longer-term sustainability of the service in Scotland. Realist

For peer review only - http://bmjopen.bmj.com/site/about/guidelines.xhtml

evaluation is well-suited to evaluating complex interventions, allowing for a theoretical and methodologically rigorous approach. In order to ascertain what works and understand how the UHVP improves the health and wellbeing of children and families in Scotland, there is the need to interrogate how contexts interact with causal forces, powers and processes that generate change. In line with a realist evaluation approach, and as outlined in figure 1, this study comprises three phases: (1) initial programme theory development; (2) programme theory validation; and (3) programme theory refinement and the development of lessons learned. Realist evaluation is methods-neutral and the use of a mixed-methods approach employing a case note review, interviews and focus groups with parents and staff, as well as parent and staff surveys, and analysis of routinely collected data, will generate and analyse both qualitative and quantitative data to achieve the goal of the evaluation. Analysis of qualitative and quantitative data will be carried out using a convergent mixed-methods approach to ensure continuous triangulation of multiple data, which will provide greater understanding of the findings in relation to policy and practice. Broadly, the evaluation could also be considered as process and outcome evaluation.

Study Setting and Population

The evaluation will be conducted over two stages and across all 14 regional Health Board areas in Scotland. It is expected that the evaluation will take four and half years to complete and the recommendations from stage one will be implemented by Health Boards prior to stage two, which will focus on examining how these recommendations have been implemented, as well as assessing any associated longer term impacts of the UHVP. However, for the purpose of this protocol paper the focus will only be orientated towards stage one of the evaluation as much of the methodology outlined will be similar in stage two.

Although this evaluation is across Scotland, five case study sites, or Health Board areas, have been selected in order to conduct in-depth case note reviews and qualitative analysis to provide deeper insight into how the UHVP programme works.

Programme theory

1. Initial programme theory development

As part of the implementation of the UHVP, Evaluability Assessment workshops were conducted in 2016 to review outcomes, identify potential data sources and provide recommendations on the methodology for the evaluation[13]. The process produced a theory of change. This theory of change was designed prior to the full roll-out of the UHVP and stakeholders involved in producing this agreed that it was important for this to be reviewed further to ensure that the perspective of national and local stakeholders involved in the planning and implementation of the national roll-out were captured. As such, workshops with national decision makers and Health Board managers were conducted to review how the

For peer review only - http://bmjopen.bmj.com/site/about/guidelines.xhtml

implementation processes were carried out, and also examined how they expected the UHVP to work, and in what contexts, in order to produce anticipated outcomes. Data from the workshops were complemented by a review of key programme documents, which were used to develop the initial programme theory. This initial programme theory informed the rest of the evaluation.

2. Programme theory validation

The initial programme theory will be tested to determine how the UHVP was implemented and delivered in practice and to assess how, as well as which outcomes were achieved. In terms of validating and refining the programme theory, four different elements spanning both qualitative and quantitative methods were identified: qualitative evaluation; case note review; surveys; and routine data analysis. As depicted in figure 2, although the evaluation will be Scotland-wide and cover all 14 Scottish Health Boards, the qualitative and case note review element will only cover five selected Health Boards or case study sites. The case study sites have been selected based on self-completion questionnaire that was distributed to Directors of Nursing at each Health Board in Scotland to enquire about the stage of UHVP implementation. Overall, the main criteria for case study site selection were (i) geographical, population and deprivation factors, (ii) UHVP implementation progress and, (iii) compatibility of local data systems with evaluation outcomes.

<Figure 2>

Qualitative research

Participants

Interviews and focus group will be conducted with health visitors who deliver the UHVP and parents in receipt of the pathway.

Health Visitors

Recruitment and Sampling

All health visitors from each study site will be sent information about the evaluation and invited to participate in either a focus group or individual semi-structured interview. Individual semi-structured interviews will be conducted with 10 health visitors from each study site in stage one. A further six participants from each study site will be involved in focus groups (approximately 80 health visitors in total). The mix of individual interviews and focus groups will help to produce rich qualitative data as important issues identified during the individual interviews can be discussed further in focus groups to understand how they are

For peer review only - http://bmjopen.bmj.com/site/about/guidelines.xhtml

challenged or supported in a group context. This will enable us to draw a more balanced contextual comparisons regarding how different Health Boards are implementing and delivering the UHVP across Scotland. Focus groups and interviews will last 45 minutes to an hour and will be audio-recorded then transcribed verbatim.

Parents

The five case study sites will allow for more in-depth examination and understanding of families' experiences of the UHVP. However, given that a number of factors such as, deprivation, rurality, vulnerability, or families identified as requiring additional support, might affect parents' experiences of the UHVP, it is important that these differences are captured through purposive sampling. This will provide better understanding of various contextual conditions across sub-groups.

In terms of data collection, parents will be recruited via their health visitors, who will inform them about the study and offer them a study information pack. The information pack will include details of how to contact the researchers if they are interested in participating in the study. Potential participants will then be contacted by one of the researchers to arrange a suitable venue and time for interview. The stage one topic guide will be informed by the initial programme theory and it is likely to include questions around families' expectations and experiences of the UHVP; what worked well for them, what did not work and why they thought that was; as well as perceived impact on how the service has influenced their health and wellbeing. High street store vouchers worth £20 will also be offered to families in return for their time. It is intended that approximately 12 individual semi-structured interviews will be conducted within each study site (60 parents overall). Informed consent will be sought to ensure anonymity of all study participants. All interviews will be audio recorded and transcribed verbatim with permission from each participant.

Qualitative Analysis – Interviews and focus groups

Qualitative data will be coded and analysed by thematic analysis, using QSR NVivo 11. Thematic analysis is particularly suited to exploring qualitative data of this nature, as it is possible to examine both within case and cross-case themes. Three key concepts - context, mechanism and outcome - of realist evaluation will drive the coding process. Two members of the research team will independently analyse the data by selecting the appropriate segments of text and code them appropriately. A final coding frame will be produced after comparison and discussion of the initial coding with the wider research team. The final coding frame will be applied to all data items. Further, similar codes will be grouped together to form overarching themes. The analysis will then advance to the interpretative phase, where potential differences in experience related to implementation, delivery and perceived impact

For peer review only - http://bmjopen.bmj.com/site/about/guidelines.xhtml

of the UHVP on outcomes will be assessed across participants' characteristics. Particular attention will be paid to how causal mechanisms of the UHVP have been influenced by contextual factors within the study sites to produce intended and unintended outcomes. This will be contextualised in terms of implementation stages for each Health Board.

Case note review

The case note review will complement health visitors' perceptions around the practice of implementing the UHVP. To ensure a focus on initial programme theory is maintained, a tool will be created and tested before data collection commences which will not request identifiable personal information in order to maintain anonymity of data gathered.

Sampling

Within each of the five case study sites health visitor managers will be approached to support the sampling of a selection of current health visitor case notes. Fifteen case notes will be sought from each of the five sites, evenly spread from the regional geographical area, providing a total of 75 case notes for review. As each of the Health Boards will be at different stages of UHVP implementation the age ranges of eligible children may vary across each area; however, to ensure that the 75 case notes identified will each provide meaningful data for analysis the following criteria, in table 1, will be applied.

Table 1. Inclusion and exclusion criteria for case note review

Inclusion criteria	Exclusion criteria
Case notes will:<br>▪ Relate to children who are at least 13 months old at the time of data collection<br>▪ Initially be selected randomly; however, purposive sampling may be required to ensure that the proportion of vulnerability in those identified is reflective of the geographical area	Case notes will **not**:<br>▪ Relate to children who have transferred into the area from another Health Board area at any time<br>▪ Relate to children whose parents have participated in the Family Nurse Partnership

Analysis

Data gathered will focus on the level of adherence to the UHVP identifying: how and when strengths and concerns around well-being were assessed; what interventions were implemented; when and where referrals were made to; and the contribution that health visitors have made to partnership working to support improving outcomes for babies,

For peer review only - http://bmjopen.bmj.com/site/about/guidelines.xhtml

children and families. The data will be analysed descriptively and compared and contrasted with findings from the other elements of the evaluation.

A note on COVID-19

It is important to mention that data collection for both the qualitative and case note review elements of the evaluation has just been completed and was not affected by the ongoing COVID-19 pandemic. How COVID-19 has affected the survey and routine data elements have been explained in their respective sections below.

National survey of health visitors and parents

Two online surveys will be employed, one with parents and one with health visitors. The surveys will provide large-scale, quantitative data on parents' experiences of health visiting and health visitors' experiences and perspectives on the UHVP that are not available through the other elements of the evaluation (e.g. routine data or case note review). Phase 1 surveys will serve as a baseline for outcomes achieved by the UHVP as it becomes more embedded in phase 2. However, the change in sampling approach (discussed below) necessitated by COVID-19 limit the extent to which the Phase 1 survey can provide a true baseline.

The survey components of the evaluation will be designed around measures relating to the initial programme theory. The surveys will include questions designed to gather the information on how the UHVP is influencing outcomes from the perspective of parents and health visitors. The questions will be designed to measure change, for example, by using scales (e.g. strongly agree, tend to agree etc.), rather than binary yes/no questions. Further information regarding the questions that will be covered in the survey is available in supplementary file 1. The survey will be conducted across all fourteen Scottish Health Boards in order to gain an overall picture of the implementation of the UHVP.

Sampling

Survey of Health visitors

Given the relatively small population of health visitors and managers [14], it is proposed that a census of all health visitors and mangers across Scotland will be conducted. A set response rate cannot be guaranteed but based on expert opinion of survey response rates amongst the public sectors in the UK, it is anticipated that this method could achieve a response rate of approximately 35% for a survey of this type. Based on the total number of health visitors in Scotland this would estimate around 600 responses to the survey. While it would be desirable to analyse the results of the survey by health board, the extent to which this could be achieved is dependent on the response rate from each health board. In the case that numbers are too low, health boards will be grouped according to a range of factors (e.g. stage of rollout, or location) and analysed on that basis.

For peer review only - http://bmjopen.bmj.com/site/about/guidelines.xhtml

Survey of Parents

With regards to the parent survey, the sample size will be based on requiring a large enough sample to facilitate disaggregation by, for example, age of child. Originally the parents survey sample frame was being drawn by local NHS Boards, however due to the strain placed on the NHS during the COVID-19 pandemic, this approach was deemed no longer viable. However, approval has been gained to use the Scottish Household Survey and Scottish Health Survey (application under consideration) re-contact database to contact families with children under 5 years old to invite them to participate. Participation will be entirely voluntary. It is likely that the new approach to sampling may reduce the number of responses we receive.

Weighting

Corrective weights will likely be applied to compensate for imbalances in the achieved profile of the survey population even if cases where the sample is reasonably representative. This will ensure that when comparing results from the two evaluation stages, the 'same' population will be compared as much as possible. Therefore, the aim of weighting will be to correct for any under- or over-representation of different groups of health visitors and parents as a result of non-response.

Analysis of Survey Data

A range of statistical techniques, depending on the nature of the data and relevancy to the evaluation will be employed. It is expected that for each question, the proportion of respondents within each sub-group giving a particular response will be analysed. Statistically significant difference between sub-groups will be examined, including difference between health visitors working in Health Boards with differing stages of implementation, differences by rurality and deprivation and age of child.

Routine data analysis (RDA)

Routine data, captured via health or social work records, or workforce data, will be used to explore the implementation of the UHVP across Scotland, and its impact on relevant outcomes among pre-school children. This is a complicated task, due to having to take account of the pathway being introduced in different Health Boards in various ways, thus meaning that children in different Health Boards become exposed at different time points. Routine data items to be explored in both the process and outcome analyses were informed by the logic model, and then refined through discussions with the Scottish Government and the Research Advisory Group, and explorations around data quality and availability.

Overall, stage one data will explore the implementation and outcomes of children prior to the UHVP being introduced, and stage two will attempt to capture changes and impact after the

For peer review only - http://bmjopen.bmj.com/site/about/guidelines.xhtml

introduction of the UHVP. All data will be examined by Health Boards, and, in addition, where possible, data will be explored by deprivation at Scotland-level. Statistical analysis plans will be written for both the process evaluation and the outcome evaluation.

Process data

The routine data analysis component of the process evaluation will address the following specific research questions:

1. What is the extent to which additional staff have been recruited to health visiting teams to support delivery of the UHVP, and associated changes in indicators of staff wellbeing such as absence and turnover rates?
2. What is the extent to which the universal child health review elements of the pathway are being delivered, the equity of these contacts, and the extent to which contacts vary by Health Board?
3. What is the extent to which child and family needs are being identified in a timely manner?

Stage 1 process data will capture information about the workforce (e.g. staff numbers, turnover, absence rates and students), coverage of the core visits, and information about those visits e.g. where they took place, who carried out the review, and which developmental assessment tools were used and how many developmental concerns were raised at different time points. This will be accessed as aggregate data through NHS Public Health Scotland. Data will be described in terms of patterns seen over time and by Health Boards and Scottish Index of Multiple Deprivation. The same data will be accessed in Stage two as in stage one, but at an individual level. This will allow for more detailed analysis of changes since UHVP was implemented in each area, as well as allowing for the calculation of yield of (i) new health plan indicators and (ii) developmental concerns raised following specific reviews, for example. Data will therefore be accessed through the electronic data research and innovation service (eDRIS) team by completing an application, which will be assessed by the Public Privacy and Benefit Panel. Data will be accessed remotely through the NHS National Safe Haven.

We will explore data related to the implementation of the UHVP intervention (the process evaluation) using graphical methods.

Outcome Data

The routine data analysis component of the outcome evaluation will contribute to this by addressing the following specific research questions:

1. What impact has implementation of the UHVP had on outcomes of children aged up to 3 years relating to;
    a. Parental health related behaviours?

For peer review only - http://bmjopen.bmj.com/site/about/guidelines.xhtml

b. Child development?
c. Child physical health?
d. Child safety?
2. How does impact vary in relation to deprivation?

The logic model items and specific outcome measures used in order to answer the research questions are presented in Table 2.

Table 2. Outcome measures in the Routine Data Analysis

**Logic model item**	**Specific outcome measures**
Improved health behaviours within families (e.g. smoke free homes, breastfeeding, weaning and early diet, oral health)	Parental smoking<br>Primary carer current smoker at 27-30m<br>Child exposed to second hand smoke at 27-30m<br>Infant feeding<br>Exclusive breast milk feeding at 6-8w<br>Any breast milk feeding at 6-8w<br>Immunisations<br>Complete coverage of universal primary and end infancy immunisations by 2nd birthday<br>Dental care<br>Any attendance at dentist by 2nd birthday
Improved child development and school readiness	Developmental concerns<br>Any developmental concern at 27-30m<br>Any concern about speech, language and communication development at 27-30m<br>Any concern about social and emotional development at 27-30m
Improved health outcomes for children and families (e.g. healthy child weight, reduced hospital admissions for serious injuries, increase in smoke-free homes, reduced substance misuse)	Child BMI<br>Child at risk of overweight or obesity (BMI ≥85th centile) at 27-30 months<br>Child clinically obese (BMI ≥98th centile) at 27-30 months<br>Unintentional injuries<br>Any hospital admission for unintentional injury by 3rd birthday<br>Any hospital admission for unintentional poisoning, burn, or scald by 3rd birthday<br>Any hospital admission for unintentional long bone fracture or head injury by 3rd birthday
Improved child safety and protection	Child protection interventions<br>Placed on child protection register at any point between birth and 3rd birthday<br>Placed on child protection register for ≥6 months between birth and 3rd birthday<br>'Looked After Child' status at any point between birth and 3rd birthday<br>'Looked After Child' status for ≥6 months between birth and 3rd birthday
Reduced inequalities in outcomes and reduced impact of wider inequalities (e.g. changing parents approach to parenting despite inequalities)	Examine all outcomes in relation to deprivation (SIMD quintile)

For peer review only - http://bmjopen.bmj.com/site/about/guidelines.xhtml

Stage one of the outcome RDA will provide baseline data on the listed measures up until implementation of the UHVP. As with the process data at stage one, this will be accessed as aggregate data through NHS Public Health Scotland. Stage 2 will provide individual participant data for children born on or after the date that the UHVP was implemented in the health board in which those children were born. Data quality will be checked.

The outcome evaluation for the RDA is a natural experiment, assessing the impact of the UHVP on the chosen outcome measures. This involves comparing the occurrence of the outcomes of interest among cohorts of children exposed to the UHVP compared to those that were unexposed to the pathway. We will use an interrupted time series (ITS) design to evaluate the intervention, since this study design is particularly suitable for evaluating the effectiveness of public health interventions. The ITS model consists of data from the unexposed children, data from children who were born on or after the UHVP intervention was implemented (the fully exposed cohort), and the counterfactual, which will be modelled by extrapolating the pre-intervention trend (data from the unexposed cohort) into the post-intervention timeline.

To provide reassurance that our analyses would be able to detect a meaningful and credible level of impact of the UHVP on the outcomes of interest, we undertook formal testing of statistical power to confirm we had adequate (≥90%) power to detect a modest (up to 20% relative) change in the majority of specific outcome measures, based on current data available on the respective outcome measures and numbers of exposed and unexposed children. Only data included in routine data collections can be used which limits the outcomes that can be analysed within this part of the evaluaton.

At present, it is unknown what effect COVID-19 will have on data collection and reporting for the latter part of this data collection.

Phase 3. Programme theory refinement

The phase will involve summarising our refined programme theory based on the findings from phase 2 to robustly articulate how mechanisms of the UHVP unfolded or did not unfold in practice, whilst identifying alternative mechanisms and explanations.

IMPLICATIONS OF THE STUDY FINDINGS

The findings of this evaluation will provide an understanding of how the UHVP works and certainly evidence the impact of increased investments in health visiting for children and families across Scotland. Finally, in line with realist evaluation methodology, this evaluation will provide recommendations on how the universal health visiting service can be improved to ensure efficient delivery of the universal health visiting pathway as well as improving outcomes for children and families, whilst reducing health inequalities. The findings will be

For peer review only - http://bmjopen.bmj.com/site/about/guidelines.xhtml

prepared as reports to the funders (Scottish Government) and presented at conferences. It will also be submitted for publication in peer-reviewed journals.

Ethical approval

This protocol has been approved by the School of Health in Social Science Research Ethics Committee, University of Edinburgh. In addition, the case note review has received approval from the Public Benefit and Privacy Panel for health and social care in Scotland. The survey has also received approval from the Scottish Government Public Benefit and Privacy Panel. Approval for the routine data analysis element of the evaluation will be sought from the Public Benefit and Privacy Panel for health and social care in Scotland.

References

1. Marmot M, Bell R. Fair society, healthy lives. *Public Health* 2012;126 Suppl 1:S4-10.
2. Geddes R, Haw S, Frank J. Interventions for Promoting Early Child Development for Health: An environmental scan with special reference to Scotland. *Scottish Collaboration for Public Health Research and Policy,* 2010. Available: http://www.scphrp.ac.uk/wp-content/uploads/2014/03/1454-scp_earlyyearsreportfinalweb.pdf [Accessed 17 June 2020]
3. NHS Health Scotland. Evidence summary: Interventions to support parents, their infants and children in the early years (pregnancy to 5 years), 2012. Available http://www.healthscotland.com/uploads/documents/19955-ParentingInterventionsPregnancyTo5Years%20amended%20151112.pdf [Accessed 17 June 2020].
4. Cowley S, Frost M. The Principles of Health Visiting: Opening the Door to Public Health Practice in the 21st Century. Amicus/Community Practitioners' and Health Visitors' Association 2006:3
5. Hoskins RAJ, 2009. Health Visiting—The end of a UK wide service? *Health policy* 2009;93(2-3):93-101.
6. Chief Nursing Directorate, Scottish Government. Public Health Nursing Services – Future Focus, CEL 13, 2013. Available: http://www.sehd.scot.nhs.uk/mels/CEL2013_13.pdf [Accessed 17 June 2020]
7. Williams L, Rycroft-Malone J, Burton CR. Bringing critical realism to nursing practice: Roy Bhaskar's contribution. *Nursing Philosophy*, 2017;18(2):e12130
8. Doi L, Jepson R, Hardie S. Realist evaluation of an enhanced health visiting programme. *PLoS ONE*, 2017;12(7):e0180569.
9. Greenhalgh T, Humphrey C, Hughes J, MacFarlane F, Butler C, Pawson R How Do You Modernize a Health Service? A Realist Evaluation of Whole-Scale Transformation in London. *Milbank Quarterly,* 2009;87:391–416.
10. Pawson R, Tilley N. Realistic evaluation. London: Sage 1997
11. Scottish Government. Child and maternal health, 2018. Available: https://beta.gov.scot/policies/maternal-and-child-health/family-nurse-partnership/ [Accessed 15 June 2020]

For peer review only - http://bmjopen.bmj.com/site/about/guidelines.xhtml

12. Scottish Parliament. Children and Young Peoples (Scotland) Act 2014, 2014. Available: http://www.legislation.gov.uk/asp/2014/8/pdfs/asp_20140008_en.pdf. [Accessed 15 June 2020]
13. NHS Health Scotland. Evaluability assessment of the enhanced health visiting pathway in Scotland, 2016. Available: http://www.healthscotland.com/documents/28090.aspx [Accessed 15 June 2020]
14. ISD Scotland, NHS Scotland Workforce Information 2017. Available: https://www.isdscotland.org/Health-Topics/Workforce/Publications/data-tables2017.asp [Accessed June 2020]

Competing interest

The authors declare that they have no competing interests.

Funding and acknowledgements

This study is being funded by the Scottish Government (Case ref: 362846). At the start of the project, LD was employed by the Scottish Collaboration for Public Health Research and Policy, which was funded by the Medical Research Council (MR/KO 023209/1) and the Chief Scientist Office. The views expressed in this publication are those of the authors and not necessarily those of the funders.

Patience and Public involvement

This study has an advisory group which includes members of the public.

Figure legend

Figure 1. Conceptual framework and data sources

Figure 2. Overview of study settings and populations in accordance with evaluation components.

For peer review only - http://bmjopen.bmj.com/site/about/guidelines.xhtml

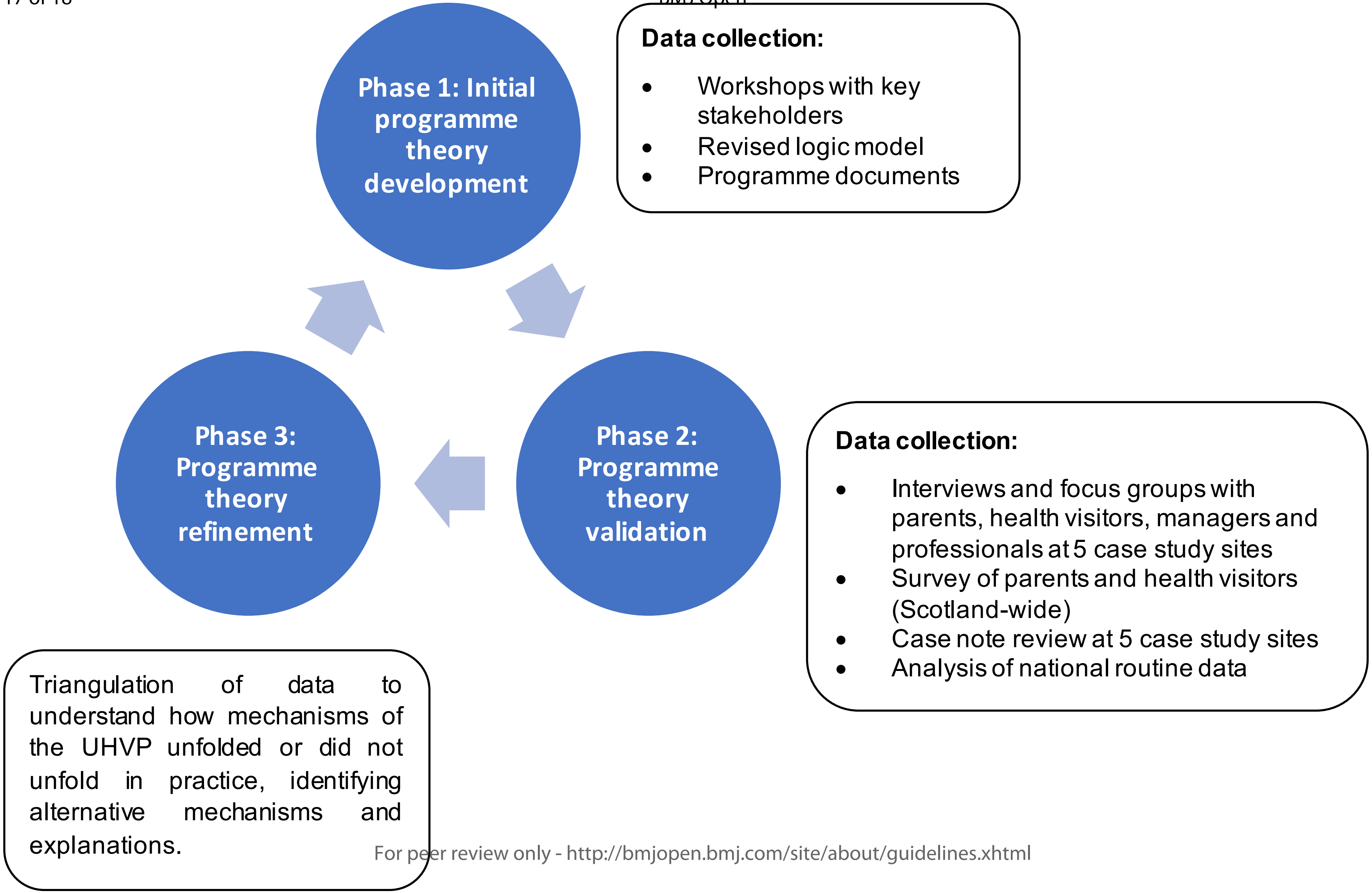
Phase 1: Initial programme theory development
Data collection:
• Workshops with key stakeholders
• Revised logic model
• Programme documents
Phase 2: Programme theory validation
Data collection:
• Interviews and focus groups with parents, health visitors, managers and professionals at 5 case study sites
• Survey of parents and health visitors (Scotland-wide)
• Case note review at 5 case study sites
• Analysis of national routine data
Phase 3: Programme theory refinement
Triangulation of data to understand how mechanisms of the UHVP unfolded or did not unfold in practice, identifying alternative mechanisms and explanations.

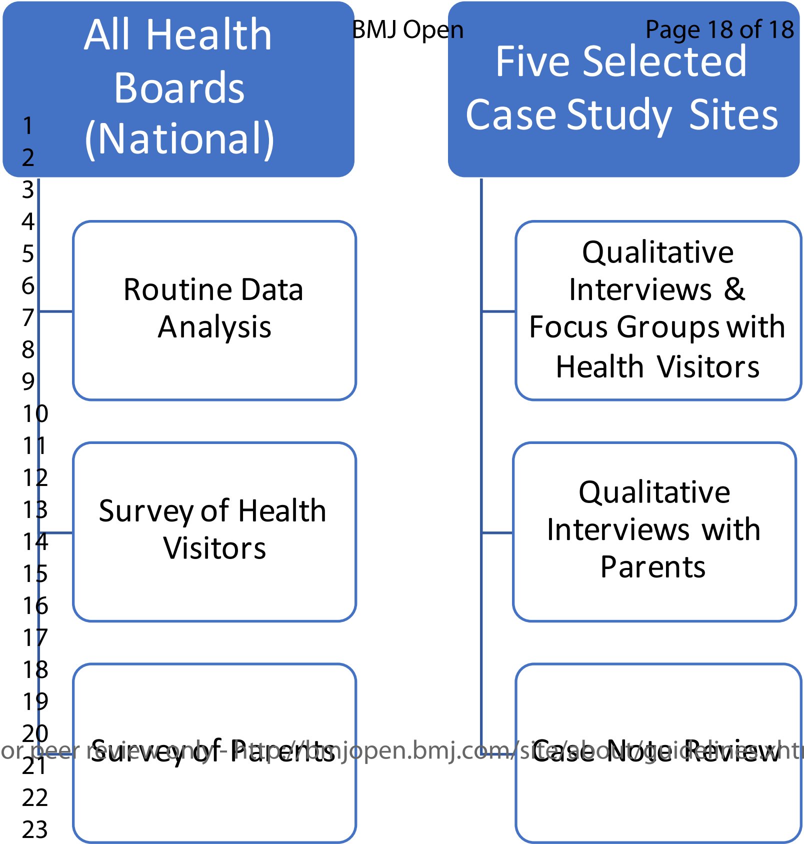

For peer review only - http://bmjopen.bmj.com/site/about/guidelines.xhtml

Supplementary file

Supplementary file 1. Health visitor and parent questionnaire topics

Health Visitor Questionnaire Topics
• The UHVP implementation process<br>• Overall views on the UHVP – including any added value and unintended consequences<br>• Training (views on the quality, relevance, frequency, gaps and CPD opportunities)<br>• Confidence to deliver all aspects of the UHVP<br>• Contacts with families (e.g., whether they are delivering all visits, assessments etc.)<br>• Relationships with families (e.g., has the UHVP affected health visitors' relationships with families?)<br>• Perceptions of how well the UHVP is working in relation to the identification of concerns relating to families/children<br>• Relationships with and referrals to other services (including working collaboratively, sharing information, and awareness of wider services etc.)<br>• Working with families who have complex additional needs<br>• IT systems (e.g. do they have access to the required systems, are the systems fit for purpose, do they manage, within the constraints of their workload, to complete the required monitoring of the UHVP?)<br>• Resources for parents (e.g. do health visitors feel they have all the necessary leaflets/materials to meet the needs of parents? What are their views on the quality of information in the materials etc.?)<br>• Impact of the UHVP on topics such as: keeping children safer; child development; child health and wellbeing; and positive health behaviours among parents.
**Parent Questionnaire Topics**
• Continuity of care from their Health Visitor<br>• Perceptions of their relationship with their Health Visitor<br>• What topics they have covered with their Health Visitor?<br>• How much they feel they know about specific health/childcare issues?<br>• Additional services their Health Visitor has suggested<br>• Confidence in own parenting<br>• Suggestions for improving the health visiting service<br>• Demographics – household structure, working status, gender, age, income, SIMD (Scottish Index of Multiple Deprivation) profile, indication of urbanity or rurality

For peer review only

Study protocol: A mixed-methods realist evaluation of the Universal Health Visiting Pathway in Scotland

Journal:	*BMJ Open*
Manuscript ID	bmjopen-2020-042305.R1
Article Type:	Protocol
Date Submitted by the Author:	28-Oct-2020
Complete List of Authors:	Doi, Lawrence; The University of Edinburgh, Scottish Collaboration for Public Health Research and Policy, School of Health in Social Science<br>Morrison, Kathleen; The University of Edinburgh, Scottish Collaboration for Public Health Research and Policy, School of Health in Social Science<br>Astbury, Ruth ; University of the West of Scotland, School of Health and Life Science<br>Eunson, Jane; Ipsos MORI Scotland<br>Horne, Margaret; The University of Edinburgh, Centre for Population Health Sciences<br>Jepson, Ruth ; The University of Edinburgh, Scottish Collaboration for Public Health Research and Policy, School of Health in Social Science<br>Marryat, Louise; University of Edinburgh, Salvensen Mindroom Research Centre<br>Ormston, Rachel ; Ipsos MORI Scotland<br>Wood, Rachael; NHS Scotland National Services Division, Information Services Division; The University of Edinburgh, Child Life and Health
**Primary Subject Heading**:	Nursing
Secondary Subject Heading:	Health services research, Public health, Qualitative research, Research methods, Paediatrics
Keywords:	PUBLIC HEALTH, Community child health < PAEDIATRICS, Health policy < HEALTH SERVICES ADMINISTRATION & MANAGEMENT, Maternal medicine < OBSTETRICS

For peer review only - http://bmjopen.bmj.com/site/about/guidelines.xhtml

I, the Submitting Author has the right to grant and does grant on behalf of all authors of the Work (as defined in the below author licence), an exclusive licence and/or a non-exclusive licence for contributions from authors who are: i) UK Crown employees; ii) where BMJ has agreed a CC-BY licence shall apply, and/or iii) in accordance with the terms applicable for US Federal Government officers or employees acting as part of their official duties; on a worldwide, perpetual, irrevocable, royalty-free basis to BMJ Publishing Group Ltd ("BMJ") its licensees and where the relevant Journal is co-owned by BMJ to the co-owners of the Journal, to publish the Work in this journal and any other BMJ products and to exploit all rights, as set out in our licence.

The Submitting Author accepts and understands that any supply made under these terms is made by BMJ to the Submitting Author unless you are acting as an employee on behalf of your employer or a postgraduate student of an affiliated institution which is paying any applicable article publishing charge ("APC") for Open Access articles. Where the Submitting Author wishes to make the Work available on an Open Access basis (and intends to pay the relevant APC), the terms of reuse of such Open Access shall be governed by a Creative Commons licence – details of these licences and which Creative Commons licence will apply to this Work are set out in our licence referred to above.

Other than as permitted in any relevant BMJ Author's Self Archiving Policies, I confirm this Work has not been accepted for publication elsewhere, is not being considered for publication elsewhere and does not duplicate material already published. I confirm all authors consent to publication of this Work and authorise the granting of this licence.

For peer review only

Study protocol: A mixed-methods realist evaluation of the Universal Health Visiting Pathway in Scotland

Lawrence Doi[1]*, Kathleen Morrison[1], Ruth Astbury[2], Jane Eunson[3], Margaret Horne[4], Ruth Jepson[1], Louise Marryat[4], Rachel Ormston[3], Rachael Wood[4]

*Correspondence: larry.doi@ed.ac.uk

[1]Scottish Collaboration for Public Health Research and Policy, School of Health in Social Science, Rm 1B31, 5 Forrest Hill, University of Edinburgh, Edinburgh, EH1 2QL

[2]School of Health and Life Science, University of West of Scotland, Paisley, PA1 2BE

[3]Ipsos MORI Scotland, 4 Wemyss Place, Edinburgh, EH3 6DH

[4]Salvensen Mindroom Research Centre, Kennedy Tower, Royal Edinburgh Hospital, University of Edinburgh, Edinburgh, EH10 5HF

For peer review only

ABSTRACT

Introduction

The growing political emphasis on the early years reflects the importance of these formative years of life. Health visitors in the UK are uniquely positioned to improve health outcomes for children and families and to reduce health inequalities. Recently, there has been a policy change in Scotland in an attempt to enhance the delivery of the universal health visiting service. This study aims to examine the extent to which the enhanced universal health visiting pathway is implemented and delivered across Scotland and to assess any associated impacts.

Methods and analysis

A mixed-methods study incorporating four methodological components and uses realist evaluation as the overall conceptual framework. It comprises three phases (1) initial programme theory development; (2) programme theory validation; and (3) programme theory refinement. The programme theory validation will use interviews and focus groups data of parents and health visitors, and conduct a case note review at five study sites. It also involves a national survey of parents and health visitors and routine data analysis of existing secondary data. The analyses of the ensuing qualitative and quantitative data will be carried out using a convergent mixed-methods approach to ensure continuous triangulation of multiple data. The findings of the evaluation will provide contextually relevant understanding of how the universal health visiting pathway works and evidence the impact of increased investments in health visiting in Scotland.

Ethics and dissemination

Protocol has been approved by the School of Health in Social Science Research Ethics Committee, University of Edinburgh. Additional approvals have been granted by the Public Benefit and Privacy Panel for health and social care in Scotland for the case note review and survey elements of the evaluation. The findings will be prepared as reports to the funders and presented at conferences. It will be submitted for publication in peer-reviewed journals.

For peer review only

Strengths and limitations of this study
• This is a large study using a theory driven approach of realist evaluation to examine the implementation and delivery of a national universal health visiting pathway.<br>• The study is expected to generate contextually relevant understanding of how the universal health visiting pathway works and evidence the impact of increased investments in the programme, whilst identifying areas for improvement, which may be relevant to other similar programmes.<br>• Using mixed-methods ensures that both qualitative and quantitative data are employed to provide continuous triangulation of data, thereby enhancing the credibility of the study findings.<br>• By not using well established experimental research approaches, some may argue against the robustness of the study design.

For peer review only - http://bmjopen.bmj.com/site/about/guidelines.xhtml

INTRODUCTION

The formative early years, which lay the foundations for physical, intellectual and emotional development, have profound life-long implications for individual health and wellbeing, education, achievement and economic status[1]. Recent early years policies across the UK reflect this and have emphasised the need for greater prevention, early identification and intervention during the early-years stage (pre-birth to five years old). The early years, including child development, protection and welfare have been recognised as key public health priorities, which are crucial to reducing health inequalities across the life course[2]. Additionally, children who experience disadvantage in the early years are at a higher risk of injury, social, emotional and cognitive difficulties[3].

Health visitors have played a longstanding and vital role supporting children and families throughout the UK. Health visiting practice has been firmly rooted in public health for over 150 years, with overall goals of health promotion and disease prevention. The professional practice of health visiting comprises 'planned activities which aim to improve the physical, mental, emotional and social health and wellbeing of the population, preventing disease and reducing inequalities in health'[4]. Health visiting is a proactive service that searches for, and responds to, health needs at the individual, family, group and community level.

Since its inception, the health visiting profession has been heavily influenced by the policies of changing governments. Over the years, the pushing and pulling of various agendas have drastically altered the scope of the profession and the nature of the service it provides[5]. This is in part due to the fact the health visitors work with children, families and communities, areas where strong and often polarising political views are held. More so, health visitors are uniquely positioned to work across health and social care divides, which few other professions are able to do. This enables them to deliver a range of health promotion, intervention and illness prevention activities to individuals and families in a variety of settings.

The growing recognition for the importance of the early years, the current political emphasis on this and a growing evidence base for health visiting practice has led to large reinvestments in health visiting services across the UK. Government policies have sought to harness health visiting as a key asset to improve health outcomes for children under the age of five and to reduce health inequalities. Efforts have also been made nationally to recruit, train and employ more health visitors leading to increased recognition and re-definition of the health visiting profession as a whole.

In 2013, the Chief Nursing Officer's Directorate, Scottish Government, undertook a scoping exercise of health visiting practice in Scotland. The findings demonstrated that there was a significant degree of variation across the service in terms of assessment, resources and visiting patterns being delivered by health visitors to families in Scotland. A refocused approach to health visiting was published by the Scottish Government in 2013[6]. The changes took into account the changing policy landscape relating to the early years, children and families, and sought to ensure that workforce capability and capacity would be equipped to

For peer review only - http://bmjopen.bmj.com/site/about/guidelines.xhtml

successfully deliver these policies. Following substantial investment in the service, the Universal Health Visiting Pathway (UHVP) was introduced in 2015.

The UHVP refocuses the role of the health visitor and includes changes to caseload weighting and management; intervention delivery; education, training and resources; and visiting patterns. The aim of this study is to examine the extent to which the UHVP is implemented and delivered across Scotland and to assess any associated impacts. To achieve this, a robust mixed-methods realist evaluation proposal has been developed to understand 'what works for whom, why and in what circumstances'. Incorporating four methodological components, this paper presents a protocol describing the use and purpose of these methods over two stages of a national-level evaluation project.

METHODS AND ANALYSIS

Conceptual framework

This is a mixed-methods study that uses realist evaluation as the overall conceptual framework to examine the UHVP across Scotland. Realist evaluation is increasingly utilised to evaluate nursing and healthcare programmes because of the valuable insight it provides into how such programmes work and how they can be improved[7]. Realist evaluations are well suited to addressing the complexity of healthcare systems and are able to produce useful findings for decision makers keen to improve healthcare programmes[8]. The strength of realist evaluation is that it can draw valuable lessons about how particular conditions make particular outcomes more likely[9]. Its goal is to explain and generate knowledge of how to improve a programme. It can also provide transferable lessons that may be used by others who intend to implement a similar programme elsewhere[10]. Realist evaluation argues that in order for the findings of evaluation to be useful to decision makers, there is the need to ask the question "what works for whom, why and in what circumstances?" By asking this question, the evaluation focuses on explaining how outcomes are achieved in respect to their contexts. This is particularly important because the UHVP has been implemented in the midst of significant policy developments in maternal and child health within Scotland, including the Family Nurse Partnership programme[11] and the Children and Young People (Scotland) Act 2014, which includes Getting it Right for Every Child (GIRFEC) and Named Person policies[12]. It is important that outcomes of the evaluation are sensitive to such policy contexts.

<Figure 1>

The study will evaluate the contexts, mechanisms, and outcomes of the UHVP and make recommendations for the longer-term sustainability of the service in Scotland. Realist

For peer review only - http://bmjopen.bmj.com/site/about/guidelines.xhtml

For peer review only

evaluation is well-suited to evaluating complex interventions, allowing for a theoretical and methodologically rigorous approach. In order to ascertain what works and understand how the UHVP improves the health and wellbeing of children and families in Scotland, there is the need to interrogate how contexts interact with causal forces, powers and processes that generate change. In line with a realist evaluation approach, and as outlined in figure 1, this study comprises three phases: (1) initial programme theory development; (2) programme theory validation; and (3) programme theory refinement and the development of lessons learned. Realist evaluation is methods-neutral and the use of a mixed-methods approach employing a case note review, interviews and focus groups with parents and staff, as well as parent and staff surveys, and analysis of routinely collected data, will generate and analyse both qualitative and quantitative data to achieve the goal of the evaluation. Analysis of qualitative and quantitative data will be carried out using a convergent mixed-methods approach to ensure continuous triangulation of multiple data, which will provide greater understanding of the findings in relation to policy and practice. Broadly, the evaluation could also be considered as process and outcome evaluation.

Study Setting and Population

The evaluation will be conducted over two stages and across all 14 regional Health Board areas in Scotland. It is expected that the evaluation will take four and half years to complete and the recommendations from stage one will be implemented by Health Boards prior to stage two, which will focus on examining how these recommendations have been implemented, as well as assessing any associated longer term impacts of the UHVP. However, for the purpose of this protocol paper the focus will only be orientated towards stage one of the evaluation as much of the methodology outlined will be similar in stage two.

Although this evaluation is across Scotland, five case study sites, or Health Board areas, have been selected in order to conduct in-depth case note reviews and qualitative analysis to provide deeper insight into how the UHVP programme works.

Programme theory

1. Initial programme theory development

As part of the implementation of the UHVP, Evaluability Assessment workshops were conducted in 2016 to review outcomes, identify potential data sources and provide recommendations on the methodology for the evaluation[13]. The process produced a theory of change. This theory of change was designed prior to the full roll-out of the UHVP and stakeholders involved in producing this agreed that it was important for this to be reviewed further to ensure that the perspective of national and local stakeholders involved in the planning and implementation of the national roll-out were captured. As such, workshops with national decision makers and Health Board managers were conducted to review how the

For peer review only - http://bmjopen.bmj.com/site/about/guidelines.xhtml

implementation processes were carried out, and also examined how they expected the UHVP to work, and in what contexts, in order to produce anticipated outcomes. Data from the workshops were complemented by a review of key programme documents, which were used to develop the initial programme theory. This initial programme theory informed the rest of the evaluation.

2. Programme theory validation

The initial programme theory will be tested to determine how the UHVP was implemented and delivered in practice and to assess how, as well as which outcomes were achieved. In terms of validating and refining the programme theory, four different elements spanning both qualitative and quantitative methods were identified: qualitative evaluation; case note review; surveys; and routine data analysis. As depicted in figure 2, although the evaluation will be Scotland-wide and cover all 14 Scottish Health Boards, the qualitative and case note review element will only cover five selected Health Boards or case study sites. The case study sites have been selected based on self-completion questionnaire that was distributed to Directors of Nursing at each Health Board in Scotland to enquire about the stage of UHVP implementation. Overall, the main criteria for case study site selection were (i) geographical, population and deprivation factors, (ii) UHVP implementation progress and, (iii) compatibility of local data systems with evaluation outcomes.

<Figure 2>

Qualitative research

Participants

Interviews and focus group will be conducted with health visitors who deliver the UHVP and parents in receipt of the pathway.

Health Visitors

Recruitment and Sampling

All health visitors from each study site will be sent information about the evaluation and invited to participate in either a focus group or individual semi-structured interview. Individual semi-structured interviews will be conducted with 10 health visitors from each study site in stage one. A further six participants from each study site will be involved in focus groups (approximately 80 health visitors in total). The mix of individual interviews and focus groups will help to produce rich qualitative data as important issues identified during the individual interviews can be discussed further in focus groups to understand how they are

For peer review only - http://bmjopen.bmj.com/site/about/guidelines.xhtml

challenged or supported in a group context. This will enable us to draw a more balanced contextual comparisons regarding how different Health Boards are implementing and delivering the UHVP across Scotland. Focus groups and interviews will last 45 minutes to an hour and will be audio-recorded then transcribed verbatim.

Parents

The five case study sites will allow for more in-depth examination and understanding of families' experiences of the UHVP. However, given that a number of factors such as, deprivation, rurality, vulnerability, or families identified as requiring additional support, might affect parents' experiences of the UHVP, it is important that these differences are captured through purposive sampling. This will provide better understanding of various contextual conditions across sub-groups.

In terms of data collection, parents will be recruited via their health visitors, who will inform them about the study and offer them a study information pack. The information pack will include details of how to contact the researchers if they are interested in participating in the study. Potential participants will then be contacted by one of the researchers to arrange a suitable venue and time for interview. The stage one topic guide will be informed by the initial programme theory and it is likely to include questions around families' expectations and experiences of the UHVP; what worked well for them, what did not work and why they thought that was; as well as perceived impact on how the service has influenced their health and wellbeing. High street store vouchers worth £20 will also be offered to families in return for their time. It is intended that approximately 12 individual semi-structured interviews will be conducted within each study site (60 parents overall). Informed consent will be sought to ensure anonymity of all study participants. All interviews will be audio recorded and transcribed verbatim with permission from each participant.

Qualitative Analysis – Interviews and focus groups

Qualitative data will be coded and analysed by thematic analysis, using QSR NVivo 11. Thematic analysis is particularly suited to exploring qualitative data of this nature, as it is possible to examine both within case and cross-case themes. Three key concepts - context, mechanism and outcome - of realist evaluation will drive the coding process. Two members of the research team will independently analyse the data by selecting the appropriate segments of text and code them appropriately. A final coding frame will be produced after comparison and discussion of the initial coding with the wider research team. The final coding frame will be applied to all data items. Further, similar codes will be grouped together to form overarching themes. The analysis will then advance to the interpretative phase, where potential differences in experience related to implementation, delivery and perceived impact

For peer review only - http://bmjopen.bmj.com/site/about/guidelines.xhtml

of the UHVP on outcomes will be assessed across participants' characteristics. Particular attention will be paid to how causal mechanisms of the UHVP have been influenced by contextual factors within the study sites to produce intended and unintended outcomes. This will be contextualised in terms of implementation stages for each Health Board.

Case note review

The case note review will complement health visitors' perceptions around the practice of implementing the UHVP. To ensure a focus on initial programme theory is maintained, a tool will be created and tested before data collection commences which will not request identifiable personal information in order to maintain anonymity of data gathered.

Sampling

Within each of the five case study sites health visitor managers will be approached to support the sampling of a selection of current health visitor case notes. Fifteen case notes will be sought from each of the five sites, evenly spread from the regional geographical area, providing a total of 75 case notes for review. As each of the Health Boards will be at different stages of UHVP implementation the age ranges of eligible children may vary across each area; however, to ensure that the 75 case notes identified will each provide meaningful data for analysis the following criteria, in table 1, will be applied.

Table 1. Inclusion and exclusion criteria for case note review

Inclusion criteria	Exclusion criteria
Case notes will:<br>▪ Relate to children who are at least 13 months old at the time of data collection<br>▪ Initially be selected randomly; however, purposive sampling may be required to ensure that the proportion of vulnerability in those identified is reflective of the geographical area	Case notes will **not**:<br>▪ Relate to children who have transferred into the area from another Health Board area at any time<br>▪ Relate to children whose parents have participated in the Family Nurse Partnership

Analysis

Data gathered will focus on the level of adherence to the UHVP identifying: how and when strengths and concerns around well-being were assessed; what interventions were implemented; when and where referrals were made to; and the contribution that health visitors have made to partnership working to support improving outcomes for babies,

For peer review only - http://bmjopen.bmj.com/site/about/guidelines.xhtml

children and families. The data will be analysed descriptively and compared and contrasted with findings from the other elements of the evaluation.

A note on COVID-19

It is important to mention that data collection for both the qualitative and case note review elements of the evaluation has just been completed and was not affected by the ongoing COVID-19 pandemic. How COVID-19 has affected the survey and routine data elements have been explained in their respective sections below.

National survey of health visitors and parents

Two online surveys will be employed, one with parents and one with health visitors. The surveys will provide large-scale, quantitative data on parents' experiences of health visiting and health visitors' experiences and perspectives on the UHVP that are not available through the other elements of the evaluation (e.g. routine data or case note review). Phase 1 surveys will serve as a baseline for outcomes achieved by the UHVP as it becomes more embedded in phase 2. However, the change in sampling approach (discussed below) necessitated by COVID-19 limit the extent to which the Phase 1 survey can provide a true baseline.

The survey components of the evaluation will be designed around measures relating to the initial programme theory. The surveys will include questions designed to gather the information on how the UHVP is influencing outcomes from the perspective of parents and health visitors. The questions will be designed to measure change, for example, by using scales (e.g. strongly agree, tend to agree etc.), rather than binary yes/no questions. Further information regarding the questions that will be covered in the survey is available in supplementary file 1. The survey will be conducted across all fourteen Scottish Health Boards in order to gain an overall picture of the implementation of the UHVP.

Sampling

Survey of Health visitors

Given the relatively small population of health visitors and managers [14], it is proposed that a census of all health visitors and mangers across Scotland will be conducted. A set response rate cannot be guaranteed but based on expert opinion of survey response rates amongst the public sectors in the UK, it is anticipated that this method could achieve a response rate of approximately 35% for a survey of this type. Based on the total number of health visitors in Scotland this would estimate around 600 responses to the survey. While it would be desirable to analyse the results of the survey by health board, the extent to which this could be achieved is dependent on the response rate from each health board. In the case that numbers are too low, health boards will be grouped according to a range of factors (e.g. stage of rollout, or location) and analysed on that basis.

For peer review only - http://bmjopen.bmj.com/site/about/guidelines.xhtml

Survey of Parents

With regards to the parent survey, the sample size will be based on requiring a large enough sample to facilitate disaggregation by, for example, age of child. Originally the parents survey sample frame was being drawn by local NHS Boards, however due to the strain placed on the NHS during the COVID-19 pandemic, this approach was deemed no longer viable. However, approval has been gained to use the Scottish Household Survey and Scottish Health Survey (application under consideration) re-contact database to contact families with children under 5 years old to invite them to participate. Participation will be entirely voluntary. It is likely that the new approach to sampling may reduce the number of responses we receive.

Weighting

Corrective weights will likely be applied to compensate for imbalances in the achieved profile of the survey population even if cases where the sample is reasonably representative. This will ensure that when comparing results from the two evaluation stages, the 'same' population will be compared as much as possible. Therefore, the aim of weighting will be to correct for any under- or over-representation of different groups of health visitors and parents as a result of non-response.

Analysis of Survey Data

A range of statistical techniques, depending on the nature of the data and relevancy to the evaluation will be employed. It is expected that for each question, the proportion of respondents within each sub-group giving a particular response will be analysed. Statistically significant difference between sub-groups will be examined, including difference between health visitors working in Health Boards with differing stages of implementation, differences by rurality and deprivation and age of child.

Routine data analysis (RDA)

Routine data, captured via health or social work records, or workforce data, will be used to explore the implementation of the UHVP across Scotland, and its impact on relevant outcomes among pre-school children. This is a complicated task, due to having to take account of the pathway being introduced in different Health Boards in various ways, thus meaning that children in different Health Boards become exposed at different time points. Routine data items to be explored in both the process and outcome analyses were informed by the logic model, and then refined through discussions with the Scottish Government and the Evaluation Research Advisory Group (see further information about this in Patient and Public involvement) and explorations around data quality and availability.

For peer review only

Overall, stage one data will explore the implementation and outcomes of children prior to the UHVP being introduced, and stage two will attempt to capture changes and impact after the introduction of the UHVP. All data will be examined by Health Boards, and, in addition, where possible, data will be explored by deprivation at Scotland-level. Statistical analysis plans will be written for both the process evaluation and the outcome evaluation.

Process data

The routine data analysis component of the process evaluation will address the following specific research questions:

1. What is the extent to which additional staff have been recruited to health visiting teams to support delivery of the UHVP, and associated changes in indicators of staff wellbeing such as absence and turnover rates?
2. What is the extent to which the universal child health review elements of the pathway are being delivered, the equity of these contacts, and the extent to which contacts vary by Health Board?
3. What is the extent to which child and family needs are being identified in a timely manner?

Stage 1 process data will capture information about the workforce (e.g. staff numbers, turnover, absence rates and students), coverage of the core visits, and information about those visits e.g. where they took place, who carried out the review, and which developmental assessment tools were used and how many developmental concerns were raised at different time points. This will be accessed as aggregate data through NHS Public Health Scotland. Data will be described in terms of patterns seen over time and by Health Boards and Scottish Index of Multiple Deprivation. The same data will be accessed in Stage two as in stage one, but at an individual level. This will allow for more detailed analysis of changes since UHVP was implemented in each area, as well as allowing for the calculation of yield of (i) new health plan indicators and (ii) developmental concerns raised following specific reviews, for example. Data will therefore be accessed through the electronic data research and innovation service (eDRIS) team by completing an application, which will be assessed by the Public Privacy and Benefit Panel. Data will be accessed remotely through the NHS National Safe Haven.

We will explore data related to the implementation of the UHVP intervention (the process evaluation) using graphical methods.

Outcome Data

The routine data analysis component of the outcome evaluation will contribute to this by addressing the following specific research questions:

For peer review only - http://bmjopen.bmj.com/site/about/guidelines.xhtml

1. What impact has implementation of the UHVP had on outcomes of children aged up to 3 years relating to;
    a. Parental health related behaviours?
    b. Child development?
    c. Child physical health?
    d. Child safety?
2. How does impact vary in relation to deprivation?

The logic model items and specific outcome measures used in order to answer the research questions are presented in Table 2.

Table 2. Outcome measures in the Routine Data Analysis

Logic model item	Specific outcome measures
Improved health behaviours within families (e.g. smoke free homes, breastfeeding, weaning and early diet, oral health)	Parental smoking<br>Primary carer current smoker at 27-30m<br>Child exposed to second hand smoke at 27-30m<br>Infant feeding<br>Exclusive breast milk feeding at 6-8w<br>Any breast milk feeding at 6-8w<br>Immunisations<br>Complete coverage of universal primary and end infancy immunisations by 2nd birthday<br>Dental care<br>Any attendance at dentist by 2nd birthday
Improved child development and school readiness	Developmental concerns<br>Any developmental concern at 27-30m<br>Any concern about speech, language and communication development at 27-30m<br>Any concern about social and emotional development at 27-30m
Improved health outcomes for children and families (e.g. healthy child weight, reduced hospital admissions for serious injuries, increase in smoke-free homes, reduced substance misuse)	Child BMI<br>Child at risk of overweight or obesity (BMI ≥85th centile) at 27-30 months<br>Child clinically obese (BMI ≥98th centile) at 27-30 months<br>Unintentional injuries<br>Any hospital admission for unintentional injury by 3rd birthday<br>Any hospital admission for unintentional poisoning, burn, or scald by 3rd birthday<br>Any hospital admission for unintentional long bone fracture or head injury by 3rd birthday
Improved child safety and protection	Child protection interventions<br>Placed on child protection register at any point between birth and 3rd birthday<br>Placed on child protection register for ≥6 months between birth and 3rd birthday<br>'Looked After Child' status at any point between birth and 3rd birthday<br>'Looked After Child' status for ≥6 months between birth and 3rd birthday

For peer review only

Reduced inequalities in outcomes and reduced impact of wider inequalities (e.g. changing parents approach to parenting despite inequalities)	Examine all outcomes in relation to deprivation (SIMD quintile)

Stage one of the outcome RDA will provide baseline data on the listed measures up until implementation of the UHVP. As with the process data at stage one, this will be accessed as aggregate data through NHS Public Health Scotland. Stage 2 will provide individual participant data for children born on or after the date that the UHVP was implemented in the health board in which those children were born. Data quality will be checked.

The outcome evaluation for the RDA is a natural experiment, assessing the impact of the UHVP on the chosen outcome measures. This involves comparing the occurrence of the outcomes of interest among cohorts of children exposed to the UHVP compared to those that were unexposed to the pathway. We will use an interrupted time series (ITS) design to evaluate the intervention, since this study design is particularly suitable for evaluating the effectiveness of public health interventions. The ITS model consists of data from the unexposed children, data from children who were born on or after the UHVP intervention was implemented (the fully exposed cohort), and the counterfactual, which will be modelled by extrapolating the pre-intervention trend (data from the unexposed cohort) into the post-intervention timeline.

To provide reassurance that our analyses would be able to detect a meaningful and credible level of impact of the UHVP on the outcomes of interest, we undertook formal testing of statistical power to confirm we had adequate (≥90%) power to detect a modest (up to 20% relative) change in the majority of specific outcome measures, based on current data available on the respective outcome measures and numbers of exposed and unexposed children. Only data included in routine data collections can be used which limits the outcomes that can be analysed within this part of the evaluation.

At present, it is unknown what effect COVID-19 will have on data collection and reporting for the latter part of this data collection.

However, the current plan is that Phase 1 of the RDA, survey, case review and qualitative data collection and analysis will mostly be completed by December 2020, whilst these elements in phase 2 will be completed by December 2022.

Phase 3. Programme theory refinement

The phase will involve summarising our refined programme theory based on the findings from phase 2 to robustly articulate how mechanisms of the UHVP unfolded or did not unfold in practice, whilst identifying alternative mechanisms and explanations.

IMPLICATIONS OF THE STUDY FINDINGS

For peer review only - http://bmjopen.bmj.com/site/about/guidelines.xhtml

The findings of this evaluation will provide an understanding of how the UHVP works and certainly evidence the impact of increased investments in health visiting for children and families across Scotland. Finally, in line with realist evaluation methodology, this evaluation will provide recommendations on how the universal health visiting service can be improved to ensure efficient delivery of the universal health visiting pathway as well as improving outcomes for children and families, whilst reducing health inequalities.

ETHICS AND DISSEMINATION

This protocol has been approved by the School of Health in Social Science Research Ethics Committee, University of Edinburgh. In addition, the case note review has received approval from the Public Benefit and Privacy Panel for health and social care in Scotland. The survey has also received approval from the Scottish Government Public Benefit and Privacy Panel. Approval for the routine data analysis element of the evaluation will be sought from the Public Benefit and Privacy Panel for health and social care in Scotland.

The findings will be prepared as reports to the funders (Scottish Government). The funders are committed to publishing the full report or a summary of the report on their website, which is available to the general public. The findings will also be presented at the annual health visitors' conference in Scotland and other maternal and child health-focused academic conferences. It will also be submitted for publication in peer-reviewed journals.

References

1. Marmot M, Bell R. Fair society, healthy lives. *Public Health* 2012;126 Suppl 1:S4-10.
2. Geddes R, Haw S, Frank J. Interventions for Promoting Early Child Development for Health: An environmental scan with special reference to Scotland. *Scottish Collaboration for Public Health Research and Policy,* 2010. Available: http://www.scphrp.ac.uk/wp-content/uploads/2014/03/1454-scp_earlyyearsreportfinalweb.pdf [Accessed 17 June 2020]
3. NHS Health Scotland. Evidence summary: Interventions to support parents, their infants and children in the early years (pregnancy to 5 years), 2012. Available http://www.healthscotland.com/uploads/documents/19955-ParentingInterventionsPregnancyTo5Years%20amended%20151112.pdf [Accessed 17 June 2020].
4. Cowley S, Frost M. The Principles of Health Visiting: Opening the Door to Public Health Practice in the 21st Century. Amicus/Community Practitioners' and Health Visitors' Association 2006:3
5. Hoskins RAJ, 2009. Health Visiting—The end of a UK wide service? *Health policy* 2009;93(2-3):93-101.

For peer review only - http://bmjopen.bmj.com/site/about/guidelines.xhtml

6. Chief Nursing Directorate, Scottish Government. Public Health Nursing Services – Future Focus, CEL 13, 2013. Available: http://www.sehd.scot.nhs.uk/mels/CEL2013_13.pdf [Accessed 17 June 2020]
7. Williams L, Rycroft-Malone J, Burton CR. Bringing critical realism to nursing practice: Roy Bhaskar's contribution. *Nursing Philosophy*, 2017;18(2):e12130
8. Doi L, Jepson R, Hardie S. Realist evaluation of an enhanced health visiting programme. *PLoS ONE*, 2017;12(7):e0180569.
9. Greenhalgh T, Humphrey C, Hughes J, MacFarlane F, Butler C, Pawson R How Do You Modernize a Health Service? A Realist Evaluation of Whole-Scale Transformation in London. *Milbank Quarterly,* 2009;87:391–416.
10. Pawson R, Tilley N. Realistic evaluation. London: Sage 1997
11. Scottish Government. Child and maternal health, 2018. Available: https://beta.gov.scot/policies/maternal-and-child-health/family-nurse-partnership/ [Accessed 15 June 2020]
12. Scottish Parliament. Children and Young Peoples (Scotland) Act 2014, 2014. Available: http://www.legislation.gov.uk/asp/2014/8/pdfs/asp_20140008_en.pdf. [Accessed 15 June 2020]
13. NHS Health Scotland. Evaluability assessment of the enhanced health visiting pathway in Scotland, 2016. Available: http://www.healthscotland.com/documents/28090.aspx [Accessed 15 June 2020]
14. ISD Scotland, NHS Scotland Workforce Information 2017. Available: https://www.isdscotland.org/Health-Topics/Workforce/Publications/data-tables2017.asp [Accessed June 2020]

Authors' contribution

LD, KM, RA, JE, RJ, LM and RW jointly conceived and developed the study proposal. LD led the writing of this manuscript with contributions from KM, RA, JE, MH, RJ, LM, RO and RW. All authors read and approved the final version of the manuscript.

Funding

This study is being funded by the Scottish Government (Case ref: 362846). At the start of the project, LD, RJ were employed by the Scottish Collaboration for Public Health Research and Policy, which was funded by the Medical Research Council (MR/KO 023209/1) and the Chief Scientist Office. The views expressed in this publication are those of the authors and not necessarily those of the funders.

Acknowledgements

The authors wish to acknowledge the guidance and contributions of staff at the Scottish Government to the design of the study.

Competing interests

The authors declare that they have no competing interests.

For peer review only - http://bmjopen.bmj.com/site/about/guidelines.xhtml

Patient and Public involvement

The study has Evaluation Research Advisory Group. This currently comprises twenty people and includes representatives of the funding organisation, academics, health and care professionals, and members of public organisations around Scotland with interest in maternal and child health. They meet twice a year with the research team to discuss and provide guidance to the various aspects of the project.

Figure legend

Figure 1. Conceptual framework and data sources

Figure 2. Overview of study settings and populations in accordance with evaluation components.

For peer review only

For peer review only - http://bmjopen.bmj.com/site/about/guidelines.xhtml

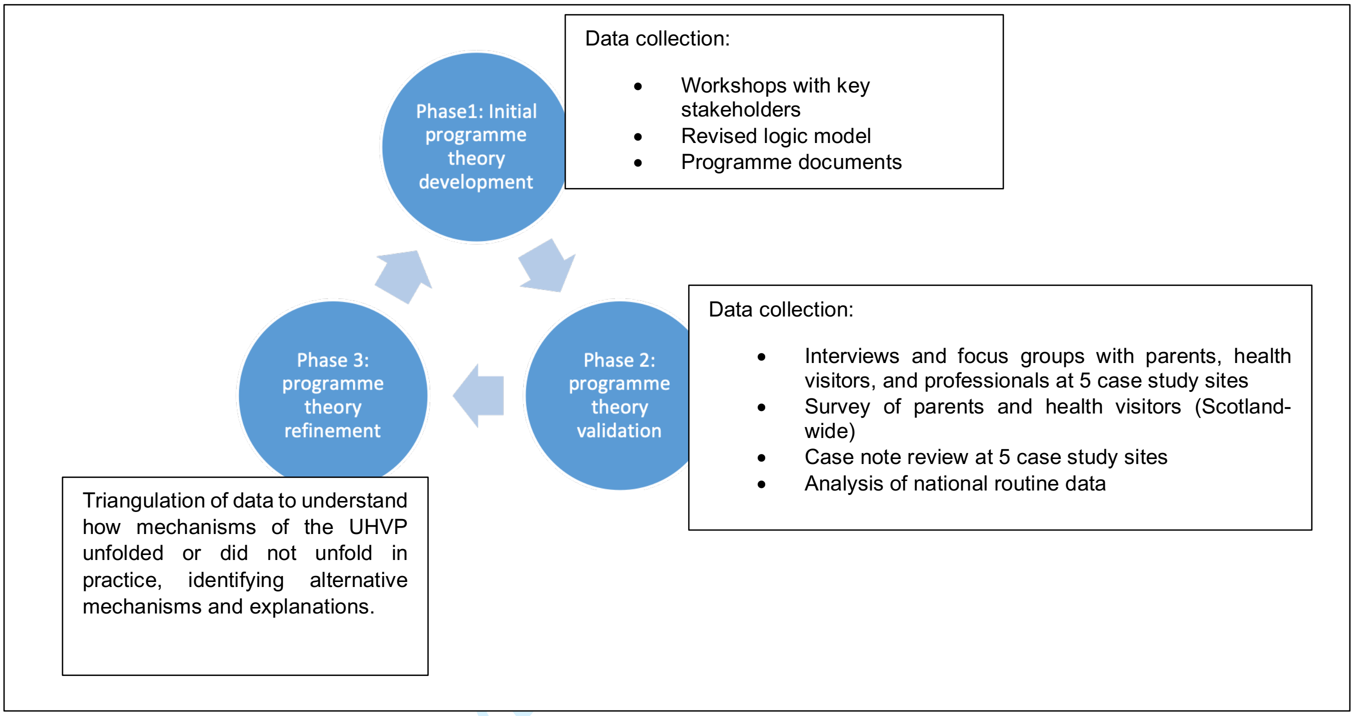

For peer review only

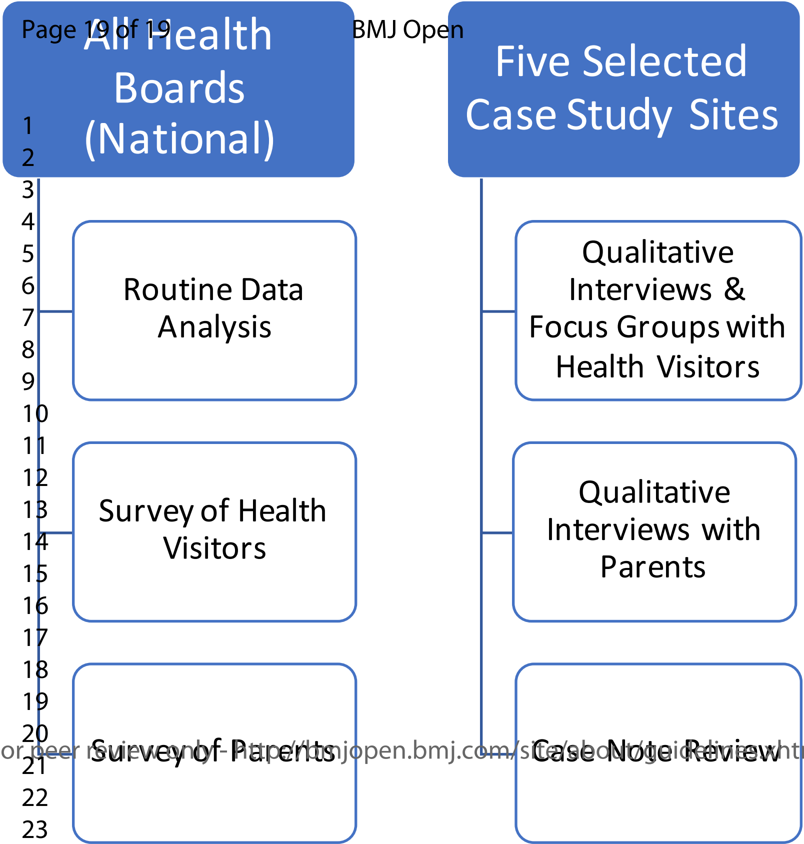

For peer review only - http://bmjopen.bmj.com/site/about/guidelines.xhtml

Supplementary file

Supplementary file 1. Health visitor and parent questionnaire topics

Health Visitor Questionnaire Topics
• The UHVP implementation process<br>• Overall views on the UHVP – including any added value and unintended consequences<br>• Training (views on the quality, relevance, frequency, gaps and CPD opportunities)<br>• Confidence to deliver all aspects of the UHVP<br>• Contacts with families (e.g., whether they are delivering all visits, assessments etc.)<br>• Relationships with families (e.g., has the UHVP affected health visitors' relationships with families?)<br>• Perceptions of how well the UHVP is working in relation to the identification of concerns relating to families/children<br>• Relationships with and referrals to other services (including working collaboratively, sharing information, and awareness of wider services etc.)<br>• Working with families who have complex additional needs<br>• IT systems (e.g. do they have access to the required systems, are the systems fit for purpose, do they manage, within the constraints of their workload, to complete the required monitoring of the UHVP?)<br>• Resources for parents (e.g. do health visitors feel they have all the necessary leaflets/materials to meet the needs of parents? What are their views on the quality of information in the materials etc.?)<br>• Impact of the UHVP on topics such as: keeping children safer; child development; child health and wellbeing; and positive health behaviours among parents.
**Parent Questionnaire Topics**
• Continuity of care from their Health Visitor<br>• Perceptions of their relationship with their Health Visitor<br>• What topics they have covered with their Health Visitor?<br>• How much they feel they know about specific health/childcare issues?<br>• Additional services their Health Visitor has suggested<br>• Confidence in own parenting<br>• Suggestions for improving the health visiting service<br>• Demographics – household structure, working status, gender, age, income, SIMD (Scottish Index of Multiple Deprivation) profile, indication of urbanity or rurality

For peer review only
